# Severe Renal Phenotype Across A Multigenerational Tuberous Sclerosis Complex (TSC) Family

**DOI:** 10.1002/mgg3.70205

**Published:** 2026-02-20

**Authors:** Elena Tuller, Joshua A. Samuels, Hope Northrup, Kate Richardson

**Affiliations:** ^1^ Division of Medical Genetics, Department of Pediatrics McGovern Medical School at UTHealth Houston, and Children's Memorial Hermann Hospital Houston Texas USA; ^2^ Division of Pediatric Nephrology and Hypertension, Department of Pediatrics McGovern Medical School at UTHealth Houston, and Children's Memorial Hermann Hospital Houston Texas USA

**Keywords:** angiomyolipoma, kidney, pathogenic variant, *TSC2*, tuberous sclerosis complex

## Abstract

**Background:**

Tuberous sclerosis complex (TSC) is an autosomal dominant genetic disorder characterized by the formation of hamartomas in the brain, kidney, and heart, along with other complex clinical manifestations, including TSC‐associated neuropsychiatric disorder (TAND). Development of genotype–phenotype correlations within TSC can aid clinicians in providing prognostic data and improve clinical management. We present here a multigenerational family who has a pathogenic variant in *TSC2* displaying a severe renal phenotype.

**Methods:**

A 23‐year‐old Caucasian male (Patient 1) was determined to have a molecularly confirmed diagnosis of TSC at approximately 2 months of age. The nonsense pathogenic variant (c.1372C>T (p.Arg458*)) in *TSC2* had been previously identified in his father (Patient 6), grandmother (Patient 5), and other extended paternal family members (Patient 2, 3, 4, 7).

**Results:**

Clinical evaluations revealed that the affected family members display a severe renal phenotype characterized by large angiomyolipoma burden (AMLs), renal cystic disease, and chronic kidney disease leading to renal failure.

**Conclusion:**

Our clinical report is of significance as it illustrates a possible genotype–phenotype correlation between a specific *TSC2* pathogenic variant and a severe renal phenotype. Our case series highlights the importance of establishing genotype–phenotype interactions to provide anticipatory guidance using prognostic data and clinical management.

## Graphical abstract

A five generation family with a known pathogenic variant (c.1372C>T; p.Arg458*) in *TSC2* displaying a severe renal phenotype characterized by large angiomyolipomas, renal cystic disease, and chronic kidney disease leading to renal failure. Our report illustrates a possible genotype–phenotype and highlights the importance of establishing genotype–phenotypes for clinical management purposes.

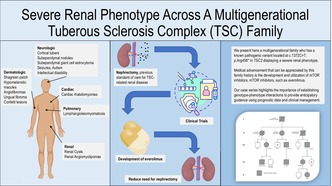

## 1 Introduction

Tuberous sclerosis complex (TSC) is an autosomal dominant genetic disorder characterized by the formation of hamartomas in the brain, kidney, and heart, along with other complex clinical manifestations, including TSC‐associated neuropsychiatric disorder (TAND). Notably, this genetic disorder exhibits both inter‐ and intrafamilial variability in clinical findings (Northrup et al. 2024). The incidence of TSC is generally estimated to be between 1:6000 and 1:10,000 live births (Northrup et al. 2021).

TSC arises from pathogenic variants in the *TSC1* (MIM: 605284) or *TSC2* (MIM: 191092) genes. *TSC1* and *TSC2* are located at 9q34.13 and 16p13.3 and encode for hamartin and tuberin, respectively. These two protein products form a heterodimer complex and act in conjunction as tumor suppressors to regulate cell growth and proliferation via the mTOR pathway (Plank et al. 1998; van Slegtenhorst et al. 1998; Han and Sahin 2011). The majority of *TSC1* and *TSC2* pathogenic variants result in loss of function. Therefore, the protein complex is inactivated leading to a loss of the inhibitory effect on the mTOR pathway, and in turn, results in the clinical phenotype of the disease (Au et al. 2004; Luo et al. 2022). mTOR inhibitors (mTORi), designed to reduce the over‐activation of the mTOR pathway, are currently FDA‐approved drugs to treat specific TSC manifestations, including subependymal giant cell astrocytomas (SEGA), renal angiomyolipoma (AML), lympangiomyoleiomatosis (LAM), and refractory epilepsy (Novartis 2021; Bissler et al. 2013, Mekahli et al. 2024).

To monitor for disease progression, there are standard management and surveillance guidelines designed for the purpose of early intervention. For neurological symptoms, brain MRIs are recommended every 1–3 years until the age of 25 years, electroencephalograms for seizures, and annual screening for TSC‐associated neuropsychiatric disorders. For renal manifestations, abdominal MRIs are to be performed every 1–3 years for progression of angiomyolipoma and cystic disease as well as assessment of renal function and blood pressure at least annually. In children, echocardiograms occur every 1–3 years until cardiac rhabdomyomas regress. Lastly, high‐resolution lung CT is to begin in biological females starting at age 18 years and to occur every 5–7 years (Mekahli et al. 2024; Northrup et al. 2021). Ideally, with the identification of TSC manifestations, clinicians can employ various interventions, such as mTORis, angiotensin‐converting enzyme (ACE) inhibitors, anti‐seizure medications, and more to treat and improve quality of life of individuals with TSC.

TSC can be diagnosed either by meeting clinical criteria and/or by harboring a pathogenic variant in either *TSC1* or *TSC2*. Because the clinical phenotype of TSC presents with significant variable expressivity, predicting the phenotype for a specific individual is nearly impossible (Curatolo et al. 2015). There are limited genotype–phenotype correlations to date relating to TSC. Pathogenic variants in *TSC2* are generally linked to a more severe phenotype and more often represent *de novo* simplex cases in comparison to *TSC1* pathogenic variants (Au et al. 2007; Peron et al. 2018). There are numerous pathogenic missense *TSC2* variants, such as p.Arg1200Trp, that are known to be associated with milder disease phenotypes (Khare et al. 2001, O’Connor et al. 2003; Mayer et al. 2004, Jansen et al. 2006, Wentink et al. 2012, Farach et al. 2017, Fox et al. 2017, Farach et al. 2017). Lastly, Zhang et al. 2021 concluded that the missense *TSC1* pathogenic variants and variants in the transcription activation domain 1 (exons 29–30) of *TSC2* led to a higher risk of renal angiomyolipoma whereas *TSC1* frameshift pathogenic variants showed a reduced risk. Developing a better understanding of genotype–phenotype correlations within TSC can aid clinicians in providing anticipatory guidance and improve clinical management. Here, we report on a multigenerational East Texas family with 10 family members who harbor a pathogenic variant (c.1372C>T (p.Arg458*)) in *TSC2* presenting with a severe renal phenotype. Our clinical report is of significance as it documents a large TSC family with a severe renal phenotype bringing to light a variant of interest.

## 2 Clinical Report

**FIGURE 1:**
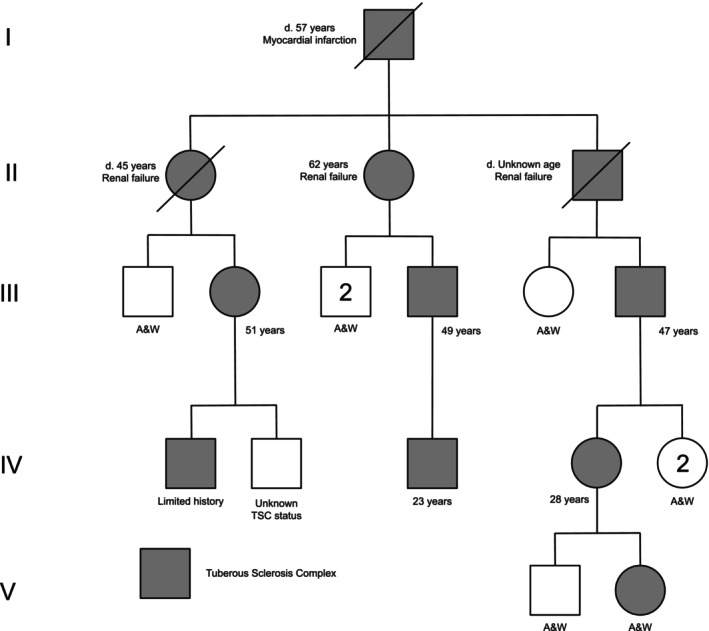
Pedigree for multigenerational family with a *TSC2* pathogenic variant demonstrates relatedness and highlights severe renal phenotype. A&W = Alive and Well; *N* = unknown if family member has TSC.

*Patient 1* (Figure 1, IV‐3) was born in 2001 after an unremarkable prenatal and birth history but had a molecularly confirmed paternal family history of TSC. Consanguinity was denied. Patient 1 was born at 42w via an emergency cesarean section secondary to non‐reassuring fetal heart tones. Neonatal course was normal. His birth weight was 4530 g (98th percentile) and birth length was 49.53 cm (41st percentile). At 3 weeks of age, Patient 1 began experiencing seizures and was evaluated by neurology. He was formally diagnosed with TSC at around 2 months of age after brain MRI revealed cortical tubers and subependymal nodules (SENs). After failing numerous anti‐epileptic drugs (AEDs), a vagal nerve stimulator (VNS) was placed at 4 years old to stabilize seizure activity with continued utilization of AEDs. Patient 1 underwent a complete TSC evaluation that analyzed the *TSC1* and *TSC2* genes in 2006 via commercial laboratory. Testing revealed a nonsense pathogenic variant (c.1372C>T (p.Arg458*)) in *TSC2* that was previously identified in his father and extended paternal family members.

Patient 1 was noted to have severe developmental delays, including speech and motor, with walking obtained at 2 years and putting words together around 10 years. He consistently obtained speech, physical, and occupational therapies throughout childhood. A formal autism evaluation was performed in early childhood that revealed Pervasive Developmental Disorder Otherwise Not Specified. Patient 1 continues to have partial symptomatic epilepsy to date and has never achieved full seizure control with his VNS and usage of polytherapy. He averages two seizures a month with his current regimen.

Patient 1 has a history of bilateral renal angiomyolipomas (AMLs) and renal cysts. At 15 years, his AMLs continued to grow, with the largest diameter noted to be 4.7 cm. In 2018, everolimus treatment was warranted to stabilize the AML growth. Patient 1 began with a 5 mg dose daily but suffered side effects including hypercholesterolemia and mouth sores, leading to a subsequent reduction in dose to 2.5 mg daily. From 2019 to 2020, the abdominal CT indicated a slight reduction in diameter from 4.2 cm to 4.1 cm. Patient 1 was diagnosed with early chronic kidney disease with an estimated glomerular filtration rate (eGFR) less than 60 mL/min. In hopes of maintaining kidney function and to achieve therapeutic dosing, Patient 1's everolimus dose was increased back to 5 mg daily in May 2021. He continued to respond well to treatment, with a reduction in diameter of his largest renal AML to 3.8 cm in November 2021, 3.1 cm in December 2022, and 3.0 cm in February 2024. His chronic kidney disease has not progressed further, and his March 2024 creatinine and cystatin C levels are stable.

*Patient 2* (Figure 1, III‐6), currently 47 years, is the paternal first cousin once removed of Patient 1. He had an unremarkable prenatal and birth history. Consanguinity was denied. Patient 2 was born at full term, and the neonatal course is unremarkable. With a known family history and consistent dermatological findings, including facial angiofibromas, ungual fibromas, and a shagreen patch, a TSC diagnosis occurred at approximately 10 years of age. Patient 2 reports normal developmental milestones but required some academic assistance. He has a history of seizures that began around 10 years and resolved around 23 years of age. There is no noted history of TAND.

Patient 2's brain MRI in July 2017 revealed cortical tubers and SENs with no evidence of a SEGA. Abdominal MRI in September 2016 revealed bilateral renal AMLs with the largest diameter measuring 8.8 cm, with noted intermittent flank pains. Patient 2 had a history of hypertension for several years with a rising creatinine level of 1.4 mg/dL. He was determined to have chronic kidney disease (CKD) stage G2/A1. He was started on valsartan‐hydrochlorothiazide in October 2016 to treat hypertension and shortly thereafter started on everolimus 5 mg daily. He was noted to be hyperlipidemic and started on atorvastatin. The everolimus dose was increased to 7.5 mg daily in June 2017 after an abdominal MRI revealed a reduction in AML diameter to 8.2 cm, but with the additional finding of bilateral renal cysts. His eGFR in April 2018 was 54 mL/min/1.73m^2^. Even with everolimus compliance, the AML increased in size, measuring 8.9 cm in January 2019 and 9.1 cm in December 2019. In conjunction with AML growth, Patient 2 complained of persistent bilateral flank pain not relieved by over‐the‐counter pain medication or warm compresses. His everolimus dose was subsequently increased to 10 mg daily. Abdominal CT in September 2021 measured the AML at 10.8 cm. Patient 2 elected to have an embolization of his left renal AML in November 2021. Due to insurance barriers, Patient 2 was lost to follow up and off everolimus for a period of time, but since resumed everolimus 10 mg daily in October 2023. An abdominal MRI performed in October 2023 revealed multiple stable left renal angiomyolipomas measuring up to 7.4 cm with stable bilateral cysts. His eGFR in August 2024 was 51 mL/min/1.73m^2^ with a Cystatin C of 1.43 mg/L.

*Patient 3* (Figure 1, II‐1), deceased at 45 years, is the paternal aunt of Patient 2. Prenatal, birth history, and neonatal course are not recalled from family members. She was diagnosed with TSC at around three to four years of age.

Dermatological findings included over 100 confetti lesions and hypomelanotic macules on extremities, one shagreen patch on the lower back, facial angiofibromas, and ungual fibromas on the hands and feet. Brain CT in 1990 showed the presence of cortical tubers with no evidence of SENs or SEGA. Seizure history included over 10 grand mal seizures, 10 partial seizures, and abnormal electroencephalogram (EEG) starting in childhood. Patient 3 received phenobarbital and phenytoin for seizure management. In adulthood, she received a formal diagnosis of sleep disturbance characterized by difficulty falling asleep and increased movement during sleep.

Per report, Patient 3 had a history of bilateral renal cysts and AMLs with the largest diameter reported as 4.0 cm. In 1989, Patient 3 underwent dialysis before receiving a kidney transplant. Patient 3 ultimately died of TSC‐related kidney complications in 1994 at 45 years of age.

*Patient 4* (Figure 1, IV‐4), currently 28 years, is the daughter of Patient 2. Prenatal history is remarkable for tobacco exposure throughout pregnancy. She was born at term via vaginal delivery and required the NICU for unspecified respiratory problems. Patient 4 reports normal developmental milestones but required some academic assistance. She underwent a complete TSC evaluation that analyzed the *TSC1* and *TSC2* genes in 2005 via a commercial laboratory. Testing revealed a nonsense pathogenic variant (c.1372C>T (p.Arg458*)) in *TSC2* that was previously identified in her father and extended paternal family members.

Dermatological findings included facial angiofibromas, ungual fibromas on fingers and toes, and one shagreen patch on the lower back. Evaluating the neurological findings, Brain MRI in February 2014 showed calcified subcortical hamartomas. Patient 4 experienced a total of three seizures shortly after a car accident in November 2015. She was not prescribed AEDs, and given the presence of head trauma, it has been reported during an encounter with a family member in 2024 that she continues to have seizures and has not sought out anti‐epileptic treatment. Chest CT in February 2014 showed no evidence of LAM.

Patient 4 also has a history of bilateral AMLs and renal cysts. Abdominal MRI in February 2014 showed multiple bilateral renal AMLs with the largest diameter noted to be 3.8 cm. Abdominal MRI in December 2016 showed that the largest AML measured 6.0 cm. She was diagnosed with early chronic kidney disease with an eGFR less than 60 mL/min. Patient 4 was started on everolimus 5 mg daily in February 2017. An updated abdominal MRI in November 2017 showed no growth of the largest AML. Patient 4 reported mouth sores and developed hypercholesterolemia as a side effect of everolimus, and she was placed on a low sodium diet for the latter. After a period of non‐compliance with everolimus, she resumed taking it daily at an increased dose of 7.5 mg daily. In January 2019, Patient 4's labs showed a therapeutic level of everolimus and abdominal MRI showed that the largest AML had decreased in size to 4.8 cm. Patient has been lost to follow up since that encounter. Patient 4's 10‐year‐old son tested negative for TSC whereas her 13‐month‐old daughter (Figure 1, V‐II) was recently tested and determined to harbor the familial variant in *TSC2*. Imaging has not been obtained yet, but she is noted to have three hypopigmented macules and currently has normal motor and speech milestones.

*Patient 5* (Figure 1, II‐2), currently 62 years, is the paternal grandmother of Patient 1. She was diagnosed with TSC around 2 years of age. Dermatological findings include more than two hypomelanotic macules, over twenty facial angiofibromas, ungual fibromas on fingers and toes, multiple shagreen patches, cephalic plaques, and confetti skin lesions. A brain MRI performed at 49 years showed SENs and cortical tubers. An ophthalmic exam performed at age 50 years noted one retinal hamartoma on the left eye.

Patient 5 has both AMLs and bilateral renal cysts. A renal ultrasound performed in January 1991 noted the presence of bilateral renal cysts. On the right kidney, the largest cyst was located on the upper pole with a small amount of hemorrhage and extrinsic compression of the cyst wall. Two smaller cysts were noted on the left kidney. Per report, Patient 5 had multiple AMLs. She is currently in renal failure and undergoing dialysis for management of chronic kidney disease.

*Patient 6* (Figure 1, III‐4), currently 49 years, is the father of Patient 1 and son of Patient 5. He was diagnosed with TSC around 16 years of age. He experienced infantile spasms until the age of one year. At age 13 years, he went into cardiac arrest. He was then found to have an undiagnosed heart murmur. Subsequent cardiac evaluation performed at age 16 years was unremarkable. Seizures began at age 16 years with 50 to 150 daily absence seizures and tonic–clonic seizures occurring one to two times annually. Brain MRI performed at age 16 years indicated multiple neurological findings consistent with TSC, including SENs and cortical tubers. Behaviorally, Patient 6 has a history of hyperactivity. At age 17, Patient 6 underwent surgery due to an unspecified urethral blockage.

Dermatological findings include more than two hypomelanotic macules, over twenty facial angiofibromas, ungual fibromas on fingers and toes, and confetti skin lesions. Additional TSC clinical manifestations include three retinal nodular hamartomas discovered during an ophthalmic evaluation performed at 27 years.

A renal ultrasound performed in January 1991 showed a small renal cyst on the right kidney. Per report, Patient 6 had one to two unilateral renal cysts and has no history of AMLs. Patient 6 currently suffers from severe paranoid schizophrenia.

*Patient 7* (Figure 1, II‐3), deceased at unknown age, is the father of Patient 2 and grandfather of Patient 6. Limited clinical information is known. In January 1991, multiple diagnostic imaging studies were performed. A renal ultrasound confirmed the presence of more than four bilateral renal cysts with no documentation of AMLs. A brain CT showed SENs and cortical tubers. Finally, an ophthalmic exam noted the presence of an achromic patch of retinal pigment epithelium in the left eye. Patient 7 died due to renal failure, although the disease progression is not known.

## 3 Discussion

Our case report demonstrates a strong renal phenotype across a multigenerational TSC family. Phenotypes not standardly observed in TSC but observed in this family include renal failure and need for dialysis.

One medical advancement that can be appreciated by this family history is the development and utilization of mTOR inhibitors. mTOR inhibitors, such as everolimus, first became FDA approved for renal AMLs relating to TSC in 2012 (Geynisman et al. 2019; Bissler et al. 2013). Older family members would not have had the opportunity to be treated with mTOR inhibitors, leading to progressive AML growth and a higher likelihood of renal failure. The family history details how everolimus has stabilized AML growth in younger family members and subsequently reduced the likelihood of future surgical intervention. The surveillance and management recommendations for TSC indicate mTOR inhibitors as a first‐line therapy for growing, asymptomatic AMLs that measure greater than 4.0 cm. For asymptomatic AMLs that are experiencing rapid growth, 3.0 cm is the minimum criteria for use of mTOR inhibitors. Acceptable second‐line treatments are embolization followed by corticosteroids, kidney resection, and ablative therapy. Additional guidelines for kidney management include abdominal MRI every one to three years, renal function assessment, and embolization followed by corticosteroids for AMLs with acute hemorrhage (Northrup et al. 2021). These comprehensive guidelines can now identify TSC‐related kidney manifestations earlier, leading to efficient intervention and positive patient outcomes.

In general, *TSC2* pathogenic variants are observed to have a more severe phenotype in comparison to *TSC1* pathogenic variants (Northrup et al. 2021). This includes a higher risk of intellectual disability, autism, infantile spasms, and renal malignancy (Kothare et al. 2014, Numis et al. 2011, Yang et al. 2014). There are few well‐described genotype–phenotype correlations, making it difficult to provide anticipatory guidance to newly diagnosed individuals. Notably, nonsense variants, such as the one observed in this family, have not been determined to produce a more severe phenotype over other variant types in TSC. Without the use of variant type in conjunction with the known intra‐ and inter‐familial variability of TSC, it becomes the clinician's responsibility to note clinical presentations that may be worthy of sharing with the larger medical community. The segregation of this variant across five generations, with adults consistently demonstrating more significant renal manifestations in comparison to the spectrum of kidney involvement known to be associated with TSC, brings this specific *TSC2* variant into consideration for genotype–phenotype correlation. Additional cases are needed to determine if a true genotype–phenotype correlation exists. Establishing additional genotype‐phenotypes in TSC can open up opportunities for clinicians to further tailor medical management for individuals with this disease.

## Author Contributions

H.N., K.R. conceptualized the study. E.T., K.R. collected the patient data and wrote the manuscript. H.N., J.S. reviewed and edited the manuscript.

## Funding

This work was supported by the UT Tuberous Sclerosis Complex Center Endowment Fund.

## Conflicts of Interest

The authors declare no conflicts of interest.

## Data Availability

The data that support the findings of this study are available on request from the corresponding author. The data are not publicly available due to privacy or ethical restrictions.
